# Dietary glycaemic index and cognitive function: prospective associations in adults of the 1946 British birth cohort

**DOI:** 10.1017/S136898001800352X

**Published:** 2018-12-26

**Authors:** Elena Philippou, Gerda K Pot, Alexandros Heraclides, Marcus Richards, Rebecca Bendayan

**Affiliations:** 1 Department of Life and Health Sciences, University of Nicosia, 46 Makedonitissas Avenue, 2417 Nicosia, Cyprus; 2 Diabetes and Nutritional Sciences Division, King’s College London, London, UK; 3 Department of Primary Care and Population Health, Medical School, University of Nicosia, Nicosia, Cyprus; 4 MRC Unit for Lifelong Health and Ageing, University College London, London, UK; 5 Department Biostatistics and Health Informatics, Institute of Psychiatry, Psychology & Neuroscience (IoPPN), King’s College London, London, UK

**Keywords:** Carbohydrates, Glucose release, National Survey of Health and Development, Prospective study, Aged

## Abstract

**Objective:**

Evidence suggests that the rate of glucose release following consumption of carbohydrate-containing foods, defined as the glycaemic index (GI), is inversely associated with cognitive function. To date, most of the evidence stems from either single-meal studies or highly heterogeneous cohort studies. We aimed to study the prospective associations of diet GI at age 53 years with outcomes of verbal memory and letter search tests at age 69 years and rate of decline between 53 and 69 years.

**Design:**

Longitudinal population-based birth cohort study.

**Setting:**

MRC National Survey for Health and Development.

**Participants:**

Cohort members (*n* 1252).

**Results:**

Using multivariable linear and logistic regression, adjusted for potential confounders, associations of higher-GI diet with lower verbal memory, lower letter search speed and lower number of hits in a letter search test were attenuated after adjustments for cognitive ability at age 15 years, educational attainment, further training and occupational social class. No association was observed between diet GI at 53 years and letter search accuracy or speed–accuracy trade-off at 69 years, or between diet GI at 53 years and rate of decline between 53 and 69 years in any cognitive measure.

**Conclusions:**

Diet GI does not appear to predict cognitive function or decline, which was mainly explained by childhood cognitive ability, education and occupational social class. Our findings confirm the need for further research on the association between diet and cognition from a life-course perspective.

As populations are ageing worldwide, nutrition research is focusing on means of maintaining normal cognitive function in old age or reducing the progression of age-related decline^(^^1^^)^. Since the human brain relies almost entirely on the oxidation of glucose^(^^2^^)^, carbohydrates are the most thoroughly studied macronutrient in this context. Specifically, evidence has been accumulating on the association between the glycaemic index (GI), a way of classifying carbohydrate-containing foods based on the rate of glucose release following consumption^(^^3^^)^, and cognitive function. Studies based on single meals differing in GI have shown that significant improvements in cognitive function occur in the late (rather than early) postprandial (i.e. after meal) phase^(^^4^^,^^5^^)^ following consumption of low-GI meals, presumably because of the more stable glucose profile at this stage. Indeed, avoiding peaks and troughs in circulating glucose is thought to be associated with better cognitive function and lower risk of cognitive impairments in the longer term^(^^6^^)^. In general, a low-GI diet, characterized by consumption of less-processed carbohydrates such as whole grains, seeded bread, pulses, legumes, fruits and nuts, has been associated with many health benefits including, but not limited to, prevention and management of diabetes, metabolic syndrome and CHD^(^^7^^)^, all of which can reduce the risk of cognitive impairment. In addition, a low-GI diet is associated with increased insulin sensitivity^(^^8^^)^, important since abnormalities in glucose regulation^(^^9^^)^ and insulin resistance are associated with a higher risk of Alzheimer’s disease and its pathogenesis^(^^10^^)^. On the other hand, consumption of a high-GI meal leads to a rapid increase in plasma glucose concentration and a concomitant high insulin response, resulting in a rapid blood glucose disposal which may cause blood glucose concentration to fall to below the fasting concentration in the late postprandial period^(^^11^^)^. Thus, dietary GI could potentially influence cognitive function in the short term through variation in the rate of glucose release, and in the long term through improvements in whole-body and perhaps brain insulin resistance.

However, most of the evidence relating GI to cognitive function stems from studies comparing the acute (i.e. within a few hours) effects of single high- or low-GI meals. To the best of our knowledge, only three observational studies have attempted to examine associations between cognitive function and diet differing in glycaemic load (GL), calculated as (GI×amount of carbohydrate)/100^(^^12^^)^, rather than using only GI. The results of studies using GL rather than GI should be assessed with caution, since it has been shown that most of the variance in diet GL stems from the amount of carbohydrate consumed rather than its quality (i.e. GI)^(^^13^^)^. Additionally, it is not possible to differentiate associations stemming from overall carbohydrate intake from those from GI itself.

Power *et al.*^(^^14^^)^ found that consumption of a high-GL diet was associated with poorer cognitive performance assessed by the Mini Mental State Examination (MMSE), which is used to screen for dementia, in a sample of community-dwelling elderly Irish (*n* 208; age range: 64–93 years). In a female cohort in Naples (*n* 1514; mean age: 56·8 (sd 4·5) years), Simeon *et al*.^(^^15^^)^ showed that GL may play a role in determining risk of cognitive impairment screened using the modified Telephone Interview for Cognitive Assessment (TICS-m) test, covering four domains: (i) orientation; (ii) registration, recent memory and delayed recall (memory); (iii) attention/calculation; and (iv) semantic memory, comprehension and repetition (language). More recently, a cross-sectional analysis of 194 cognitively healthy older adults showed that GL was inversely associated with global cognitive function and figural memory assessed using a cognitive test battery only in those with poor glucoregulation^(^^16^^)^. In the latter study, the cognitive assessment comprised seven tasks evaluating the following six cognitive domains: (i) concept formation; (ii) executive processing speed/inhibition; (iii) verbal memory; (iv) verbal fluency; (v) figural memory; and (vi) complex attention. Thus, the evidence from epidemiological studies so far is highly heterogeneous, in terms of cohorts studied and in cognitive function assessed. Furthermore, an important aspect of such analyses is that other covariates such as the metabolic syndrome^(^^17^^)^, characterized by visceral obesity, dyslipidaemia, hypertension and hyperglycaemia, should also be taken into consideration since these are associated both with diet GI^(^^18^^)^ and an increase risk of cognitive decline^(^^19^^)^. Additionally, factors related to dietary choice, such as childhood cognitive ability and adult education and socio-economic position^(^^20^^,^^21^^)^, are also linked to adult cognition, and could thus confound the association between dietary factors and cognitive ability in old age^(^^22^^)^.

The aim of the current study was to examine prospective associations between diet GI and cognitive function, using data from the Medical Research Council (MRC) National Survey of Health and Development (NSHD). The NSHD provides a unique opportunity to investigate these associations in a nationally representative birth cohort study with detailed measures of diet in midlife, a range of cognitive measures in later life, and key covariates such as childhood cognitive ability and detailed evaluation of adult socio-economic position. We hypothesized that consumption of a low-GI diet at age 53 years would be associated with better cognitive function at 69 years and with a slower decline in cognitive function from 53 to 69 years old.

## Participants and methods

### Participants

The NSHD, also known as the British 1946 birth cohort, is a socially stratified sample of 5362 individuals (2547 males and 2815 females) born during one week in March 1946 in England, Scotland and Wales^(^^23^^)^. The cohort has been followed up twenty-four times, most recently in 2015 at age 69 years, where 41·5 % of the original cohort (*n* 2226) was eligible for inclusion after exclusion of those who had died (*n* 1026), lived abroad (*n* 578), had previously refused consent (*n* 1105), were incapable (*n* 10), untraceable (*n* 37) or not contacted (*n* 380). Participation in the 24th NSHD data collection at age 68–69 years was high; the postal questionnaire, nurse visit and overall participation rates (84, 80 and 94 %, respectively) were even higher than the equivalent rates achieved at 60–64 years (81, 78 and 84 %)^(^^24^^)^. From the 2226 individuals who had responded at age 69 years, the present analysis includes 1252 participants who had complete diet data (exposure) at age 53 years and cognitive function assessment (outcome) at both ages 53 and 69 years. The sample used in the current analysis (*n* 1252) is similar in demographic characteristics to the participants responding at age 69 years^(^^24^^)^. The study has been conducted according to the guidelines laid down in the Declaration of Helsinki. Ethics approval was obtained from the North Thames Multicentre Research Ethics Committee and written informed consent was obtained from all cohort members.

### Dietary data

Dietary data collected at age 53 years were used to investigate associations with cognitive function at age 69 years. We chose to investigate diet at age 53 years for a number of theoretical and practical reasons. From a practical point of view, data from relevant covariates (e.g. measurements of blood lipids, blood pressure and use of medication) were also available at this age while a number of longitudinal studies assessing dietary patterns and cognitive decline for healthy ageing had a comparable follow-up period of 11–13 years^(^^25^^–^^27^^)^.

Details of dietary assessment of this cohort have been previously published^(^^28^^)^. In brief, dietary data were collected for five consecutive days using estimated diet diaries. For the present analysis, diet data of those who had completed at least three consecutive days were analysed. Participants were asked to record all foods and drinks consumed both at home and away using household measures. Portion sizes were estimated using guidance notes and photographs of portion sizes. Diet diaries were checked before coding and calculation of the average daily nutrient intakes was undertaken using time-appropriate nutrient databases. GI values were assigned to all foods with total carbohydrate content >0·1 g/100 g using the methodology described by Aston *et al*.^(^^29^^)^. The average GI of the diet was calculated by assigning a GL for each food item, summing the GL values for the day and dividing this by the total carbohydrates in grams^(^^30^^)^. Dietary under-reporting was assessed as previously described^(^^31^^)^. First, the ratio of energy intake (EI; derived from the food diaries) to estimated energy requirement (EER) was calculated according to an individualized method^(^^32^^)^. EER were estimated based on individual physical activity levels using equations from the Institute of Medicine of the National Academies^(^^33^^)^. A 95 % CI for EI:EER was calculated^(^^34^^)^ to account for the variability of the methods used to estimate EI and EER. The 95 % CI for the NSHD was 0·54, 1·46; thus, individuals reporting EI less than 54 % of their EER were classified as under-reporters and those reporting more than 146 % as over-reporters. Dietary variables included in the statistical models as potential confounders were total energy intake, percentage of energy from fat, saturated fat, alcohol and carbohydrate, NSP intake (g/d) and EI:EER.

### Cognitive function

Data on cognitive function at ages 69 years (outcome) and 53 years (used as a baseline covariate) were used in the present study. Cognitive function at age 69 years was chosen as the outcome of interest since cognitive decline appears to be more evident after 60 years of age^(^^35^^,^^36^^)^. Two tests at age 69 years represented the key fluid functions of memory and speed of processing. Verbal memory was assessed using a fifteen-item word-list learning task where the participant was shown each word for 2s and asked to write down as many of these from memory as possible, in any order, within 1 min. The test was repeated twice for a total of three learning trials. To minimize practice effects, parallel forms were used and alternated over successive assessments. A letter search test was used to assess mental speed, visual scanning and focused concentration. It required participants to cross out as many targets, the letters P and W, embedded in a letter matrix, as quickly and accurately as possible within 1 min. This test was scored for: number of correct hits; speed, i.e. the last target crossed out by the time limit; accuracy, calculated as [(number of correct hits)/(number of targets hit plus number of targets missed)]×100; and speed–accuracy trade-off, i.e. accuracy/speed.

### Covariables

Cognitive abilities at age 15 years, educational attainment by age 26 years, further training by age 43 years, occupational social class (SC), anthropometric, health and dietary variables (as listed above), as well as cognitive function at time of exposure (age 53 years), were included in the statistical models as potential confounding variables based on their association with both exposure and outcome variables of interest^(^^19^^,^^20^^,^^22^^,^^37^^,^^38^^)^ and their previous association with cognitive decline in this cohort^(^^39^^–^^42^^)^.

Cognitive abilities at age 15 years was assessed using the Heim AH4 test^(^^43^^)^, the Watts–Vernon reading test^(^^44^^)^ and a forty-seven-item mathematics test^(^^44^^)^. The AH4 is a 130-item ability test with verbal (analogies, comprehension and numerical reasoning) and non-verbal items (matching, spatial analysis and non-verbal reasoning) summed to yield a general ability score. The Watts–Vernon is a test of reading comprehension that requires selection of appropriate words to complete thirty-five sentences. An overall score of cognitive abilities at age 15 years was calculated as the average summary measure of the above test scores standardized to the whole population on each component test. Education was captured as the highest educational qualifications and their training equivalents attained by age 26 years and classified as: none, vocational only, ordinary secondary (‘O’ levels), advanced secondary (‘A’ levels), or degree level or equivalent. Any further training by age 43 years was classified as: training but no qualifications, training and qualifications up to ‘O’ level or equivalent, and training and qualifications at ‘A’ level or equivalent or beyond. Current or last occupational social class at age 53 years (SC) was classified according to the UK Registrar General^(^^45^^)^ as: professional, managerial and technical; skilled non-manual; skilled manual; partly skilled manual; or unskilled manual. Additionally, the following measures were taken during home visits at age 53 years by trained nurses: weight and height from which BMI was calculated; waist circumference measured at a point midway between the costal margin and the iliac crest and in line with the mid-axilla; brachial blood pressure measured twice in succession with the survey member sitting; and a non-fasting venous blood sample, from which HDL-cholesterol and TAG concentrations were assayed^(^^46^^)^. Physical activity at age 53 years was coded as inactive (no participation), moderately active (1–4 times/month) and most active (≥5 times/month) participation in sports or recreational activity. Smoking was coded as current, ex-smoker or never smoked^(^^47^^)^, and use of antihypertensive medication was coded based on interview and questionnaire data.

### Statistical analyses

Data distribution was assessed using normality plots; normally distributed variables are presented as mean and sd, and skewed variables as median and quartiles 1 and 4 (Q1–Q4). Baseline differences in cognitive function and covariates as a function of GI quartiles were examined using Pearson’s *χ*^2^ test, one-way ANOVA or the Kruskall–Wallis test. *Post hoc* analyses were carried out to further examine differences in cognitive function between GI quartiles.

Multivariable linear regression analysis tested the associations of dietary GI at age 53 years with verbal memory and letter search speed at age 69 years, adjusting for potential confounders. Considering the skewed nature of letter search number of correct hits, letter search accuracy and speed–accuracy trade-off, these were categorized into tertiles and ordinal logistic regression was carried out to explore their association with dietary GI at age 53 years with the first GI quartile (lowest GI) being treated as the reference category. The mean difference with 95 % CI and the OR with 95 % CI are presented for linear and ordinal logistic regression, respectively.

All analyses were first performed unadjusted (model 1) and then were adjusted for sex (model 2), further adjusted for cognitive abilities at age 15 years, education, training and SC (model 3), further adjusted for BMI, waist circumference, smoking status, physical activity, blood pressure, HDL-cholesterol, TAG and antihypertensive medication (model 4), and further adjusted for total energy intake, percentage of energy from fat, saturated fat, alcohol and carbohydrate, NSP (g/d) and EI:EER (model 5). To examine the association between dietary GI at age 53 years and change in cognitive measures between ages 53 and 69 years, conditional models of change incorporated corresponding cognitive measures at baseline (age 53 years; model 6). To explore possible collinearity between cognitive abilities at age 15 years, educational attainment, training and SC included in model 3, as well as to determine which of these variables could be responsible for the attenuation in the association, further sensitivity analyses were also performed.

All analyses were performed on the sample with complete data on both diet and cognitive function (*n* 1252). To reduce potential bias from missing covariables, variables with missing values (i.e. cognitive abilities at age 15 years (*n*_missing_ 173), education (*n* 61), further training (*n* 114), SC (*n* 49), BMI (*n* 3), smoking (*n* 1), physical activity (*n* 1), blood pressure (*n* 17), HDL-cholesterol (*n* 224), TAG (*n* 151) and antihypertensive medication (*n* 1)) were imputed using multiple imputation to maximize the analytical sample. Multiple imputations by fully conditional specification via Markov Chain Monte Carlo (MCMC) were performed; all analyses presented were conducted across thirty imputed data sets and combined using Rubin’s rules^(^^48^^,^^49^^)^. Sensitivity analyses comparing complete cases and imputed data were performed. Statistical analyses were performed using the statistical software package IBM SPSS Statistics for Windows version 22.0 and *P*<0·05 was considered statistically significant.

## Results

The characteristics of the NSHD study population (*n* 1252) by dietary GI quartiles are shown in Table 1. In terms of prevalence, cohort members consuming a higher-GI diet were more likely to be male, had lower cognitive abilities at age 15 years, a lower educational level by age 26 years, lower further training by age 43 years and a lower SC. They also had a higher BMI and waist circumference, were less physically active, were more likely to smoke, use antihypertensive medication, had a higher systolic and diastolic blood pressure, and had lower HDL-cholesterol and higher TAG concentrations (all *P*<0·05). Dietary intake of the cohort members at age 53 years is shown in Table 2. Participants consuming a higher-GI diet also consumed more energy, had a higher carbohydrate, sugar, starch, fat, saturated fat and alcohol intake, consumed less NSP and had a higher GL per 4184 kJ (1000 kcal) than those with a lower-GI diet (all *P*<0·001). With regard to cognitive function outcomes, those with a higher-GI diet had a lower baseline verbal memory score (*P*<0·001), as well as lower baseline letter search speed score and number of targets hit (*P*<0·01).

**Table 1:** Characteristics of the studied population by dietary glycaemic index (GI) quartile at age 53 years (*n* 1252): 1946 British birth cohort

			Dietary GI quartile								
	All participants (*n* 1252)		1st GI quartile (low) (*n* 332)		2nd GI quartile (*n* 301)		3rd GI quartile (*n* 303)		4th GI quartile (high) (*n* 316)		
	%	*n*	%	*n*	%	*n*	%	*n*	%	*n*	*P* value†
Sex (% female)	53·4	669	68·7	228	58·8	177	50·8	154	34·8	110	<0·001
Smoking (% current)	15·8	198	7·5	25	12·3	37	16·2	49	27·5	57	<0·001
SC (% professional)	9·2	115	8·7	29	13·6	41	10·2	31	4·4	14	<0·001
Education (% degree)	13·6	170	15·7	52	18·9	57	11·9	36	7·9	25	<0·001
Further training (% at ‘A’ level)	8·5	107	8·7	29	9·6	29	8·9	27	7·0	22	0·013
Physical activity (% very active)	37·9	475	45·8	152	38·9	117	36·3	110	30·4	96	<0·001
Antihypertensive medication (% yes)	12·0	150	9·0	30	10·6	32	16·5	50	12·0	38	0·028
											
	Mean or Median	sd or Q1–Q4	Mean or Median	sd or Q1–Q4	Mean or Median	sd or Q1–Q4	Mean or Median	sd or Q1–Q4	Mean or Median	sd or Q1–Q4	
Cognitive abilities age 15 years	0·26	0·78	0·40	0·76	0·37	0·76	0·24	0·78	0·05	0·79	<0·001
HDL-cholesterol (mmol/l)	1·70	0·50	1·84	0·47	1·71	0·49	1·65	0·51	1·59	0·50	<0·001
TAG (mmol/l)	2·06	1·46	1·68	1·06	1·99	1·57	2·13^b^	1·39	2·44	1·67	<0·001
Verbal memory score (age 69 years)	22·7	5·9	23·6^a^**^,b^***	5·7	23·6^c^**^,d^***	5·7	22·1^a^**^,c^**	6·2	21·3^b^***^,d^***	5·7	<0·001
Letter search speed (age 69 years)	265·3	72·4	275·1^a^*	71·9	264·9	70·5	265·6	73·9	258·2^a^*	72·4	0·022
BMI (kg/m^2^)	26·1	23·8–29·0	25·3	23·4–28·2	25·9	23·8–28·6	26·6	24·1–29·8	26·7	24·4–29·2	<0·001
Waist circumference (cm)	88·8	79·9–98·1	84·2	77·0–94·8	88·0	79·1–97·2	90·7	82·4–99·2	93·2	84·6–100·7	<0·001
Systolic BP (mmHg)	133	121–146	129	117–143	131	120–143	134	122–148	137	126–150	<0·001
Diastolic BP (mmHg)	82	75–91	80·0	74–87	82	75–90	84·0	76–92	84	78–93	<0·001
Letter search number of correct targets hit (age 69 years)	24·0	21·0–28·0	24·0^a^*^,b^***	21·0–29·0	24·0^c^*	21·0–28·0	23·0^a^*	20·0–28·0	23·0^b^***^,c^*	20·0–27·0	0·002
Letter search accuracy‡ (%) (age 69 years)	90·6	83·3–96·0	90·9	83·0–96·0	90·9	84·0–96·0	90·5	82·4–96·0	90·5	83·3–95·2	0·306
Speed–accuracy trade-off§ (age 69 years)	0·35	0·28–0·42	0·33^a^*^,b^*^,c^*	0·27–0·41	0·35^a^*	0·28–0·42	0·35^b^*	0·28–0·42	0·35^c^*	0·29–0·42	0·066

SC, occupational social class; BP, blood pressure; Q1, quartile 1; Q4, quartile 4. ^a,b,c,d^Mean values within a same row with the same superscript letters were significantly different: *P<0·05, **P<0·01, ***P<0·001.

† One-way ANOVA or Kruskall–Wallis test for continuous data or Pearson’s *χ*^2^ test for categorical data used to compare the GI quartiles. *Post hoc* comparisons done by Tukey’s test.

‡ Accuracy calculated as: [(number of hits)/(number of hits + number of missed)]×100.

§ Speed–accuracy trade-off calculated as: accuracy (see above)/speed.

**Table 2:** Dietary intake at age 53 years of the studied population by dietary glycaemic index (GI) quartile (*n* 1252): 1946 British birth cohort

			Dietary GI quartile								
	All participants		1st GI quartile (low)		2nd GI quartile		3rd GI quartile		4th GI quartile (high)		
	Mean or Median	sd or Q1–Q4	Mean or Median	sd or Q1–Q4	Mean or Median	sd or Q1–Q4	Mean or Median	sd or Q1–Q4	Mean or Median	sd or Q1–Q4	*P* value†
Total energy intake (kJ)	8431	2050	7899	1955	8435	1900	8422	1912	8996	2255	<0·001
Total energy intake (kcal)	2015	490	1888	467	2016	454	2013	457	2150	539	<0·001
Carbohydrate intake (% of energy)	44·4	7·1	46·1	7·2	45·0	5·9	44·9	7·1	41·7	7·3	<0·001
Sugar intake (% of energy)	20·3	6·0	23·4	6·1	20·7	4·9	19·9	5·7	17·0	5·6	<0·001
Starch intake (% of energy)	24·0	4·8	22·5	4·8	24·2	4·2	24·7	4·7	24·5	5·1	<0·001
Protein intake (% of energy)	15·9	2·7	16·3	3·0	15·8	2·6	15·8	2·5	15·5	2·5	<0·001
Total fat intake (% of energy)	34·2	5·9	33·0	6·1	34·3	5·8	34·3	6·0	35·2	5·7	<0·001
Saturated fat intake (% of energy)	13·4	3·3	12·8	3·3	13·3	3·3	13·4	3·3	13·9	3·3	<0·001
Alcohol intake (% of energy)	3·6	0·8–8·4	3·3	1·0–6·7	3·9	0·9–7·7	3·3	0·1–7·9	5·5	0·8–11·8	<0·001
NSP (g/d)	14·9	4·7	16·0	4·8	16·0	4·7	14·5	4·2	12·9	4·2	<0·001
EI:EER	0·9	0·2	0·8	0·2	0·9	0·2	0·9	0·2	0·9	0·2	0·001
Diet GI	61·8	4·0	56·8	2·0	60·6	0·8	63·2	0·7	66·8	1·9	<0·001
Diet GL per 8368 kJ (2000 kcal)	146·0	23·2	139·4	22·0	145·5	19·2	151·4	23·9	148·1	25·3	<0·001

EI:EER, energy intake:estimated energy requirement; GL, glycaemic load.

† One-way ANOVA or Kruskall–Wallis test for continuous data or Pearson’s *χ*^2^ test for categorical data used to compare the GI quartiles. *Post hoc* comparisons done by Tukey’s test.

Preliminary analysis found no interactions between sex and diet GI; thus results are presented for the total sample. As shown in Table 3, a higher-GI diet was associated with a lower verbal memory score and a lower letter search speed score which remained significant when adjusted for sex (model 2). These associations were fully attenuated when the models were further adjusted for cognitive abilities at age 15 years, education, further training and SC (model 3). When we additionally examined change in cognitive function between ages 53 and 69 years by including the corresponding baseline cognitive function measures (model 6), no association between diet GI at age 53 years and change in either verbal memory score or letter search speed score was found.

**Table 3:** Association between diet glycaemic index (GI) at age 53 years and cognitive function test results at age 69 years, analysed as continuous outcome variables by linear regression (*n* 1252): 1946 British birth cohort

	GI continuous					
	Verbal memory score			Letter search speed		
	Regression coefficient	95% CI	*P* value	Regression coefficient	95% CI	*P* value
Model 1	−0·26	−0·34, −0·18	<0·001	−1·65	−2·65, −0·65	0·001
Model 2	−0·22	−0·30, −0·14	<0·001	−1·39	−2·42, −0·36	0·008
Model 3	−0·01	−0·09, 0·07	0·805	−0·60	−1·67, 0·47	0·269
Model 4	0·01	−0·07, 0·08	0·897	−0·32	−1·41, 0·77	0·568
Model 5	0·01	−0·08, 0·09	0·918	−0·59	−1·75, 0·57	0·318
Model 6	0·03	−0·04, 0·10	0·370	−0·22	−1·22, 0·79	0·674

Model 1: unadjusted. Model 2: adjusted for sex. Model 3: adjusted for cognitive abilities at age 15 years, educational attainment and occupational social class. Model 4: as model 3 and further adjusted for BMI, waist circumference, smoking status, physical activity, blood pressure, HDL-cholesterol, TAG and antihypertensive medication. Model 5: as model 4 and further adjusted for energy intake, percentage of energy from fat, saturated fat, alcohol and carbohydrate, NSP intake (g/d) and energy intake:estimated energy requirement. Model 6: as model 5 and further adjusted for cognition at age 53 years.

As shown in Table 4, a higher-GI diet at age 53 years was associated with decline in the number of targets hit at age 69 years which remained significant after adjustment for sex (model 2). Similar to the above findings, the association was fully attenuated in model 3 and no association between diet GI at age 53 years and rate of change in number of targets hit between 53 and 69 years was found (model 6). No association was found between diet GI at age 53 years and letter search accuracy or speed–accuracy trade-off at age 69 years.

**Table 4:** Association between diet glycaemic index (GI) quartiles age 53 years and cognitive function test results at age 69 years, analysed by ordinal logistic regression† (*n* 1252): 1946 British birth cohort

		2nd GI quartile		3rd GI quartile		4th GI quartile (high)		
	1st GI quartile (low)	OR	95 % CI	OR	95 % CI	OR	95 % CI	*P* value for trend
Letter search number of targets hit								
Model 1	1·00 (ref.)	0·88	0·66, 1·18	0·79	0·60, 1·05	0·65	0·49, 0·87	0·001
Model 2	1·00 (ref.)	0·91	0·68, 1·21	0·84	0·63, 1·12	0·72	0·53, 0·96	0·012
Model 3	1·00 (ref.)	0·84	0·60, 1·16	0·93	0·66, 1·29	0·92	0·65, 1·29	0·188
Model 4	1·00 (ref.)	0·86	0·62, 1·20	0·99	0·71, 1·39	0·97	0·68, 1·38	0·727
Model 5	1·00 (ref.)	0·84	0·60, 1·17	0·94	0·67, 1·32	0·90	0·62, 1·31	0·466
Model 6	1·00 (ref.)	0·82	0·58, 1·16	0·87	0·61, 1·25	0·93	0·63, 1·39	0·658
Letter search accuracy								
Model 1	1·00 (ref.)	1·08	0·81, 1·44	0·94	0·71, 1·25	0·88	0·67, 1·17	0·183
Model 2	1·00 (ref.)	1·11	0·83, 1·48	0·97	0·73, 1·30	0·95	0·71, 1·27	0·405
Model 3	1·00 (ref.)	1·23	0·82, 1·58	1·14	0·82, 1·58	1·12	0·80, 1·59	0·760
Model 4	1·00 (ref.)	1·15	0·83, 1·60	1·20	0·86, 1·67	1·14	0·81, 1·64	0·632
Model 5	1·00 (ref.)	1·17	0·84, 1·63	1·23	0·88, 1·73	1·26	0·86, 1·83	0·464
Model 6	1·00 (ref.)	1·09	0·78, 1·53	1·13	0·81, 1·60	1·21	0·83, 1·77	0·515
Letter search speed–accuracy trade off								
Model 1	1·00 (ref.)	1·28	0·96, 1·71	1·21	0·91, 1·62	1·24	0·94, 1·65	0·106
Model 2	1·00 (ref.)	1·27	0·95, 1·69	1·19	0·89, 1·59	1·20	0·90, 1·61	0·181
Model 3	1·00 (ref.)	1·33	0·96, 1·85	1·17	0·84, 1·62	1·13	0·80, 1·60	0·499
Model 4	1·00 (ref.)	1·30	0·94, 1·82	1·11	0·80, 1·54	1·07	0·75, 1·52	0·728
Model 5	1·00 (ref.)	1·34	0·96, 1·87	1·18	0·84, 1·65	1·16	0·80, 1·69	0·443
Model 6	1·00 (ref.)	1·18	0·84, 1·66	1·05	0·74, 1·48	1·00	0·68, 1·47	0·976

Ref., reference category. Model 1: unadjusted. Model 2: adjusted for sex. Model 3: adjusted for cognitive abilities at age 15 years, educational attainment and occupational social class. Model 4: as model 3 and further adjusted for BMI, waist circumference, smoking status, physical activity, blood pressure, HDL-cholesterol, TAG and antihypertensive medication. Model 5: as model 4 and further adjusted for energy intake, percentage of energy from fat, saturated fat, alcohol and carbohydrate, NSP intake (g/d) and energy intake:estimated energy requirement. Model 6: as model 5 and further adjusted for cognition at age 53 years.

† For ordinal regression, outcome variables were categorized into tertiles.

Sensitivity analysis were performed including each covariate in model 3 independently (cognitive abilities at age 15 years, education, training and SC) for each cognitive outcome. For verbal memory score, the results showed that the attenuation in the association between verbal memory score and GI seen in model 3 was confounded by cognitive abilities at age 15 years (*B*=−0·05; 95 % CI −0·12, 0·03; *P*=0·22) and education (*B*=−0·04; 95 % CI −0·12, 0·04; *P*=0·28), independently. Adjusting for further training (*B*=−0·20; 95 % CI −0·29, −0·12; *P*<0·001) or SC (*B*=−0·14; 95 % CI −0·22, −0·06; *P*<0·001), independently, did not attenuate the association between diet GI and verbal memory score. Similar results were found for letter search speed, where cognitive abilities at age 15 years (*B*=−0·88; 95 % CI −1·93, 0·18; *P*=0·10) and education (*B*=−0·67; 95 % CI −1·74, 0·39; *P*=0·22) each independently attenuated the association between diet GI and search speed, but not further training (*B*=−1·31; 95 % CI −2·35, −0·28; *P*=0·01) and SC (*B*=−1·11; 95 % CI −2·16, −0·07; *P*=0·03). With regard to the number of hits, cognitive abilities at age 15 years (*B*=0·99; 95 % CI 0·96, 1·02; *P*=0·43), education (*B*=0·99; 95 % CI 0·96, 1·02; *P*=0·34) and SC (*B*=0·98; 95 % CI 0·95, 1·01; *P*=0·13), each independently attenuated the association between diet GI and number of hits, while further training did not (*B*=0·97; 95 % CI 0·94, 1·00; *P*=0·05).

Further sensitivity analyses including the cognitive function variables as *Z*-scores showed very similar results to the ones presented. Similarly, results using complete case analysis (*n* 931) did not differ substantially from the presented results based on imputed data.

## Discussion

In the current prospective analysis examining associations between dietary GI at age 53 years and cognitive function at age 69 years in the 1946 birth cohort, it was shown that a higher-GI diet at age 53 years was associated with lower scores in verbal memory, letter search number of hits and letter search speed, after controlling for sex. These associations were attenuated when adjusted for cognitive abilities at age 15 years, educational attainment and SC. Previous work in the same cohort found that a consistently healthy dietary choice at ages 36 and 43 years was associated with slower memory decline and letter search speed at age 60 years^(^^39^^)^. That specific study, however, found an association of an overall healthy dietary choice based on consumption of breakfast, type of milk and bread, and number of fruits and vegetables with cognitive function, and thus did not differentiate which of these individual dietary factors were associated with cognition. To the best of our knowledge, the present analysis is the first to specifically investigate prospective associations between dietary GI, a measure of carbohydrate quality with potential effects on rate of glucose release^(^^11^^)^, and cognitive function over later adulthood when cognitive decline is more evident, and lends further support to the possibility of such an association being confounded by childhood cognitive ability and adult-life socio-economic position (primarily educational attainment and to a lesser extent SC).

Similar to these findings, in analyses of the 1936 Lothian Birth Cohort study, Corley *et al*.^(^^50^^)^ found that a ‘Mediterranean-style’ diet was associated with better, and a ‘traditional’ diet (high in tinned vegetables, meat pies, custard sauces, milk-based puddings, and drinking less filter, espresso or cappuccino coffee) with poorer, cognitive function. These associations, however, were mostly attenuated after adjustment for childhood IQ and adult SC (as in the current study) and only small associations persisted between diet and verbal ability only.

The present findings are not surprising due to increasing evidence associating intelligence and education with health and survival^(^^21^^,^^51^^)^. Indeed, previous work in the 1946 birth cohort showed that childhood cognition was associated with a healthy dietary choice at age 53 years although this association (including that of exercise) could not be fully explained by education^(^^21^^)^. These effects can be interpreted in a number of ways. As demonstrated by Richards *et al*.^(^^21^^)^, childhood cognitive ability might have a lasting effect on adult dietary choice. Childhood cognitive abilities and education are related to healthier food choice (a low-GI diet in the present case), and in turn better cognitive function, through increased knowledge about nutrition^(^^52^^)^. Moreover, a higher childhood cognitive ability predicts advantageous social circumstances in adulthood including financial circumstances and SC^(^^53^^)^. It is indeed well established that those who are disadvantaged financially and/or socially are more likely to be less health conscious and have a lower health literacy and self-management of health^(^^54^^)^, including perhaps their risk of cognitive decline^(^^55^^)^. A lower SC is also associated with consumption of foods of lower nutritional value and lower-quality diets that cost less per kilojoule but are poorer in health-protective nutrients^(^^20^^)^, such as whole grains and fruit which have a low GI. Indeed, in the present study it was shown that those with lower cognitive abilities at age 15 years, lower educational attainment and lower SC consumed a higher-GI diet. Thus the findings of the present study add to the existing evidence^(^^50^^)^ to support an interactive cycle involving cognitive function, self-management of health (including diet) and cognitive outcomes as proposed by Anstey *et al*.^(^^56^^)^. Additionally, education shapes the individual by increasing confidence, motivation and self-regulation^(^^57^^)^; all of which are important for self-management of health. It is of interest that even in the Whitehall II Study, a white-collar middle-aged cohort, significant associations observed between dietary patterns and cognitive deficit were considerably attenuated when the models were adjusted for education^(^^22^^)^. This confounding arises from the fact that education is associated with dietary choice^(^^21^^)^ and is an important predictor of verbal cognitive ability, even when childhood cognitive ability is controlled for^(^^41^^)^.

Based on the above, our findings add to the existing literature on the importance of taking into consideration childhood cognitive ability, education and SC when investigating associations between diet and cognitive function. This will allow better understanding of the extent by which dietary patterns or specific dietary factors explain cognitive variation in old age over and above that explained by earlier cognitive ability. Indeed, not all studies investigating associations of diet GI or GL with cognitive function have taken account of all the above possible confounders. In the study by Power *et al.*^(^^14^^)^ in community-dwelling older Irish adults, where consumption of a low-GL diet was found to be associated with poorer MMSE scores, there was no adjustment for childhood cognitive ability or education, and SC was only partly captured by data on residential property price. In the EPIC-Naples women cohort^(^^15^^)^, cognitive status was positively associated with diet GL and negatively associated with education, but the association of diet GL and cognitive function controlling for education was not assessed, neither was childhood cognitive ability or SC taken into account in any of the analyses. Lastly, in the Brain in Motion study^(^^16^^)^, statistical models were adjusted for the overall intellectual level using results of the North American Adult Reading Test (NAART) but it seems that the NAART results were used interchangeably with education. As discussed above, however, the association(s) of childhood cognitive ability and education with nutrition may be independent or synergistic. It is evident that, overall, the aforementioned cohort studies reporting associations between diet GL and cognitive function did not adequately adjust for previous cognitive ability, education and SC. Furthermore, as discussed above, the cognitive function tests used and the domains assessed in previous studies were highly heterogeneous, which may have led to disparity in findings.

The present study has a number of strengths. First, the NSHD is a life-course cohort drawn from the general UK population and the current analysis is the first to assess the prospective associations between dietary GI and cognitive function. Due to the longitudinal design of the NSHD, it provided the unique opportunity to address the potential confounding roles of childhood cognitive function, educational and occupational attainments, as well as other measures, when examining these associations. Another key strength is that assessment of cognitive function captured a number of cognitive domains (i.e. episodic memory, mental speed, visual scanning and focused concentration) using sensitive tests. Additionally, the availability of measures of cognitive function at both ages 53 and 69 years enabled us to study prospectively the associations of diet GI and cognition in older individuals. Moreover, unlike most cohort studies which rely on FFQ for dietary assessment, the NSHD uses food diaries^(^^28^^)^, which allow for a much more detailed assignment of dietary GI values to individual foods (rather than food groups) and do not rely on dietary recall which is memory-dependent and would potentially result in bias especially in those with memory issues. Prospective diet diaries also have a significantly better correlation with intake biomarkers and less regression dilution than FFQ^(^^58^^)^. Nevertheless, we cannot exclude the possibility that participants may temporarily change their diet during the period of recording, leading to information bias. A limitation of the current study is the unavoidable issue of sample attrition which is common in prospective cohorts^(^^59^^)^. It is possible that more health-conscious or healthier cohort members are more likely to remain in the study. Indeed, the study had a disproportionate loss to follow-up of those who were socio-economically disadvantaged and had lower cognitive ability in childhood^(^^60^^)^. Lastly, it would be interesting to assess whether the associations between diet GI and cognitive function vary by the participants’ status of glucose tolerance and/or insulin resistance, but these measurements were not available at age 53 years in our study. Nevertheless, we did not find any preliminary associations between diet GI or cognitive function and diabetes status, a finding that could be attributed to the small sample of participants with diabetes in this sample; and thus further research should focus on diabetic populations.

## Conclusion

In conclusion, the current prospective study showed that dietary carbohydrate quality, assessed using the GI, does not appear to be a key factor in predicting cognitive function or the potential decline over the age 53 to 69 years; which was largely explained by childhood cognitive ability, education and adult social class. Our findings confirm the need for further research on the association between diet and cognition from a life-course perspective.
